# Ecological Succession, Hydrology and Carbon Acquisition of Biological Soil Crusts Measured at the Micro-Scale

**DOI:** 10.1371/journal.pone.0048565

**Published:** 2012-10-30

**Authors:** Matthew Tighe, Rebecca E. Haling, Richard J. Flavel, Iain M. Young

**Affiliations:** School of Environmental and Rural Science, University of New England, Armidale, New South Wales, Australia; Estacion Experimental de Zonas Áridas (CSIC), Spain

## Abstract

The hydrological characteristics of biological soil crusts (BSCs) are not well understood. In particular the relationship between runoff and BSC surfaces at relatively large (>1 m^2^) scales is ambiguous. Further, there is a dearth of information on small scale (mm to cm) hydrological characterization of crust types which severely limits any interpretation of trends at larger scales. Site differences and broad classifications of BSCs as one soil surface type rather than into functional form exacerbate the problem. This study examines, for the first time, some hydrological characteristics and related surface variables of a range of crust types at one site and at a small scale (sub mm to mm). X-ray tomography and fine scale hydrological measurements were made on intact BSCs, followed by C and C isotopic analyses. A ‘hump’ shaped relationship was found between the successional stage/sensitivity to physical disturbance classification of BSCs and their hydrophobicity, and a similar but ‘inverse hump’ relationship exists with hydraulic conductivity. Several bivariate relationships were found between hydrological variables. Hydraulic conductivity and hydrophobicity of BSCs were closely related but this association was confounded by crust type. The surface coverage of crust and the microporosity 0.5 mm below the crust surface were closely associated irrespective of crust type. The δ ^13^C signatures of the BSCs were also related to hydraulic conductivity, suggesting that the hydrological characteristics of BSCs alter the chemical processes of their immediate surroundings via the physiological response (C acquisition) of the crust itself. These small scale results illustrate the wide range of hydrological properties associated with BSCs, and suggest associations between the ecological successional stage/functional form of BSCs and their ecohydrological role that needs further examination.

## Introduction

Biological soil crusts (BSCs) are widespread in semi-arid and arid zones globally [1], [2] and may comprise large areas of the non-vegetated soil surface [3]. Several classifications of BSCs exist which predominantly relate to disturbance sensitivity and/or stages of ecological succession [2], [4], [5]. These types of classifications are consistent with site observations, and generally follow the sequence of cryptomorphic filamentous cyanobacteria, to early successional/perimorphic bryophytes, followed by the more hypermorphic byrophytes [2]. The relationship between these classifications and the hydrological properties of BSCs are still largely unexplored [2], due in part to the difficulties in quantifying hydrological properties of these surfaces.

Hydrological properties of BSCs are difficult to measure *in-situ*. Studies commonly report on hydrological properties such as runoff at a reasonably large scale (for example, following rainfall simulation over 1 m^2^ plots [6]). Runoff values of BSCs at this scale are extremely variable and findings often difficult to place in a wider context [7], [8], [9]. This is due to several factors; (i) BSCs are often classified and examined as one type of surface cover, irrespective of the range of functional forms present, (ii) there is a lack of information on hydrological properties of different functional crust forms at a small scale (mm) which compound the difficulties in interpreting hydrological responses at larger scales, (iii) previous studies often only compare crusts with soils that have been disturbed (scalped, trampled, burnt, and (iv) different methodologies have been applied in different studies [2], [10]. These factors have also caused difficulties in a wider context – with BSCs often covering large areas of the soil surface, the inability to determine hydrological responses of this surface ‘type’ makes hydrological interpretations of a whole site ambiguous. In particular, BSCs are variably considered as both hydrophilic and hydrophobic and as potentially having high or low porosity, while other hydrological properties such as hydraulic conductivity or potential for waterlogging are only rarely considered [2], [11], [12].

The variable surface coverage of BSCs at both large and small scales makes hydraulic conductivity of any one type of BSC difficult to quantify, while the relationship of different crust types with inundation or surface saturation may be almost impossible to determine without long term observation of a site or experimental manipulation [11]. Measurement of equivalent pore diameter via permeameter readings is also difficult due to the variable hydrophobic/hydrophilic nature of BSCs [13]. Recently Menon et al. [14] used X-ray tomography to directly measure macroporosity of crusted surfaces after careful removal and transportation of the intact surfaces. These authors modeled water infiltration and showed how the larger pore sizes (>50 µm) greatly affected water flow in the cyanobacteria and cyanobacteria-lichen dominated crusts they examined. This approach of undertaking a series of measurements on carefully transported, undisturbed BSCs has potential in measuring several hydrological variables under controlled laboratory settings and subsequently determining any relationships these variables have with each other, or with crust type.

In addition to the possibility of moving samples into a controlled laboratory setting, differences in the carbon isotopic signature of BSCs could potentially be used to indicate relative inundation of surfaces. BSCs that have a history of frequent inundation may have an elevated (or more positive) carbon isotope signature than crusts that have been inundated relatively less frequently. However, this potential relationship has not been examined in a semi-arid environment [11], [15].

In this study we examine a range of functionally different biological crusts taken from one site in semi-arid south eastern Australia. This study focuses on the porosity and small-scale hydrological properties of different crusted surfaces to determine if there are distinct differences in hydrological properties of the crust types sampled, including C and δ ^13^C signatures. The aim of this investigation was to ascertain if there were differences in the measured hydrological properties of the different crusts that could account for the wide range of hydrological interpretations made in association with BSCs. A secondary aim of this study was to ascertain if any of these hydrological properties could be directly related to the common classification of BSCs based on disturbance sensitivity/ecological succession.

## Methods

### Site Description

**Figure 1:**
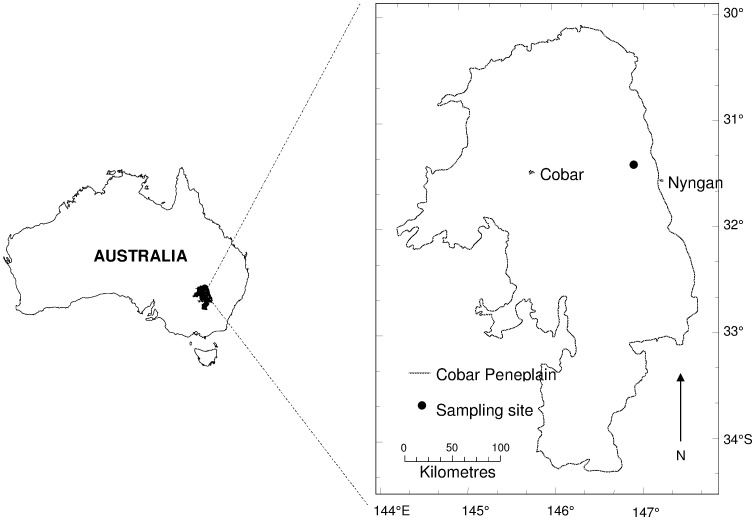
Location of the sampling site in southeastern Australia. The site is located in the Cobar Peneplain bioregion in western NSW, Australia. Nearby regional population centres are also depicted (modified from [38]).

Biological crusts were collected at one privately owned site approximately 30 km northwest of Nyngan in western NSW, Australia (Figure 1). No permits were necessary for this field study. Permission for the study to be undertaken was granted by the owner of the private land, Mr. A. Gibson. The long-term average annual rainfall at the site is 446 mm, distributed evenly throughout the year. The site was in a mid-slope position and was comprised of a dense *Geijera*, *Dodonaea* and *Eremophila* sp. shrubland with a scattered over-storey of *Eucalytpus populnea* and *E. intertexta*. Soil was a Red Kandosol [16] or a Chromic Luvisol in the FAO classification.

The site had minimal herbaceous plant cover and clumped woody debris and litter cover, and a wide range of biological crust cover occurring both in inter-canopy areas and underneath woody canopies (Figure S1). Grazing history of the site was typical of the region, with set stocking of 0.5–1.5 DSE over 12–18 month periods common. Previous sampling and analysis at the site [9] indicated that surface soil texture was very consistent (sand, silt and clay % means and standard errors of 70.1±0.26, 13.4±0.16, 16.5±0.13; n = 6).

### Soil Core Collection

Seven different soil surface types (A through G) were identified and sampled (Table 1) within an area of 50 m×50 m. Three replicates of each surface type were collected, except for sample G where only two examples of the surface type could be found (Table 1, n = 20). The first replicate of each sample was taken as the first of each type encountered during a haphazard search of the area. Subsequent replicates of each type were selected following random distance and orientation walks which were repeated until two more of each surface type were encountered (with the exception of G). Sampling consisted of manually inserting a 5 cm diameter, 10 cm in height zinc alloy cylinder into the soil until the rim of the cylinder was 1–2 mm above the highest point of the soil surface. The intact core and surface was then removed using a hand trowel, wrapped in foam packaging, placed in a foam lined container, and transported to the laboratory. All coring occurred under field moisture conditions (<7% moisture w/w) and samples were left for two weeks to air dry in the laboratory before analysis. Crusts were monitored visually over the whole analysis period to ensure no obvious detrimental effects due to drying or disturbance were observed.

**Table 1:** Dominant crust type and hydrological properties for each sample^1^. Numerical values are arithmetic means ±1 standard error^2^.

Variable	Filamentous cyanobacteria – unidentified (A)	Filamentous cyanobacteria – unidentified (B)	Crustose lichen – *Diploschistes* spp. (C)	Liverwort/squamulose lichen mix – *Riccia* spp. and *Cladonia* spp. with minor unidentified filamentous cyanobacteria (D)	Liverwort – *Riccia* spp. (E)	Moss – *Bryum* spp. (F)	Foliose lichen – *Xanthoparmelia* spp. (G)
Sensitivity to disturbance 3	Low	Low	Mid	Mid to High	High	High	High
Successional stage^3^	Early	Early	Mid	Mid to Late	Late	Late	Late
Percent cover	55.9±5.78bc	24.5±2.71a	82.1±3.16c	34.1±2.36ab	49.8±8.28ab	50.6±a1.44bc	35.2±17.6ab
WDPT (s)	3.6±2.47a	5.9±1.19ab	64.4±16.8b	36.3±29.7b	3.8±1.21a	4.7±1.45a	>1200c
Sorptivity@ 0 cm head (mm h ^−1/2^)	NA	0.04±0.008a	0.07±0.03a	0.05±0.03a	0.07±0.03a	NA	NA
Hydraulic conductivity (mm h^−1^)	1.00±0.37bc	0.47±0.07ab	0.09±0.04ab	0.93±0.26bc	2.30±0.83c	4.53±0.43d	0.00d
Porosity 0–5 mm (%)	13.7±0.91a	20.7±3.49a	13.1±1.28a	12.2±1.36a	13.7±2.56a	14.0±3.49a	13.8±0.61a
Porosity 0.5–1 mm (%)	18.1±3.95a	20.5±6.67a	10.5±0.84a	18.9±0.44a	23.7±4.25a	18.2±3.90a	25.1±2.70a
C 0–5 mm (%)	1.94±0.14ab	1.40±0.18a	1.95±0.06ab	1.62±0.10a	2.95±0.45bc	3.29±0.21c	2.54±0.65abc
δ ^13^C 0–5 mm	−23.4±0.10ab	−22.5±0.23b	−22.5±0.37b	−23.0±0.05ab	−23.2±0.06ab	−24.0±0.23a	−23.2±0.82ab

1 Means followed by different letters indicate significant differences within a row.

2 N = 3 for each sample except for cover calculation of sample B (n = 2) and all variables for sample G (n = 2).

3 As indicated by Belnap [2], Eldridge and Greene [4] and Eldridge and Tozer [5].

### Crust Identification

Crusted surfaces were identified to genus where possible but were placed in a functional group according to life form at a minimum, as per Eldridge and Tozer [5]. Crusts were also classified into groups as related to their sensitivity to disturbance and their approximate appearance in successional progression at a site [2], [4], [5]. The percent surface area of the biological crust of each sample was calculated using high resolution colour photographs (RGB) and manual adjustment of auto-thresholding in ImageJ following conversion to a binary black and white image, and using the original image as a reference for any manual thresholding adjustment [17]. Cyanobacteria surfaces were inspected both dry and following surface wetting to check for any differences in cover (no differences evident). One replicate of the filamentous cyanobacteria (sample B) was found to be extremely difficult to threshold (dry or wet) and the resulting cover value appeared as an obvious outlier in analyses. This value was omitted from statistical analysis.

### 3-dimensional Measurements

All intact cores were scanned using a high-resolution Vtomexs X-ray Computed Tomography (CT) system (GE Phoenix). The use of µX-ray tomography for the measurement of soil porosity is now established [18]. The source was a 240 kV ‘Direct’ tube with a reflection tungsten target and spot size of 4 µm. Preliminary trials indicated optimal energy settings were 130 kV and 110 µA with a 0.5 mm thick copper filter to reduce beam hardening artifacts and all scans were acquired using these settings. A series of 750 angular projections (radiographs) were obtained in a 360° revolution, each the average of 3, 400 ms exposures captured on a 512×512 pixel detector array.

The projections were reconstructed into 16-bit volumes with an isotropic voxel size of 100 µm using GE Phoenix proprietary software on a graphical processor cluster. The volumes were then converted to a stack of.tiff images using VG Studio Max (Version 2.0) and imported into Image J (Version 1.46i for 64-bit Windows 7) for further analysis. The volumes were then converted to 8-bit grey scale values (required for thresholding algorithms), and the centermost 250×250 pixels (equivalent of 25 mm×25 mm) of each slice were selected using a pixel mask. The ‘Equalize histogram’ algorithm inside the ‘Enhance Contrast’ tool was used to improve the efficacy and consistency of thresholding. Several representative slices from different depths of the cores were isolated and several ‘Automatic Threshold’ algorithms were applied which indicated that the ‘Yen’ automatic threshold [19] was most representative of the pore space in the scans and was consequently applied to all image stacks. The binary images were analysed using SCAMP (Version 1.2) [20], [21] to firstly determine the porosity by slice for each stack. Due to the uneven soil surface, the surface was determined to be the first slice where porosity was less than 40%. This selection was visually inspected to confirm the appropriate delineation of the surface. From this digital slice downward porosity, pore space connectivity and pore size distribution measures were obtained for the remaining volume (approx. 430 slices) using SCAMP and the data was summarised using Excel 2010 for Windows 7 (64-bit).

### Hydrological Properties

Water repellency of each crusted surface was determined with the water drop penetration test (WDPT) [22]. Six drops were placed on the surface of each intact core using a syringe, ensuring that drops were only placed on the surface of the identified dominant crust. A video recording of each test was made and the time taken for each water drop to penetrate the surface (to the nearest second) was determined from video playback. The average time of penetration was calculated for each sample replicate from the six values. Sample G showed no penetration of water drops before the termination of the test at 1200 s, and the average time for water penetration for sample G was taken as 1200 s.

A custom made microinfiltrometer [23] was used to determine short term (up to 10 min) infiltration of each intact crust surface. The infiltrometer tip (internal diameter 1.61 mm) was placed in contact with the dominant crust identified in each sample replicate and the infiltration (calculated from changes to water weight in a reservoir) was recorded at 10 second intervals. Sorptivity and hydraulic conductivity (the initial, rapid, non-linear rate of water absorbance by a surface and the longer term rate of water movement through a surface respectively) at 0 cm head were estimated using Philip’s [24] equation:
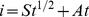
where i = infiltration, S = sorptivity, t = time in seconds, and A = hydraulic conductivity

Infiltration measurements before 50 seconds duration were omitted due to high random variation in early data points, as per the recommendation of Smiles and Knight [25]. Data were graphed as t^1/2^ versus it^−1/2^ with sorptivity and hydraulic conductivity calculated from the intercept and slope of the least squares linear regression. For samples where the average sorptivity of the replicates could not be differentiated from zero a value was not reported. For sample G no infiltration occurred within 10 minutes, with the corresponding sorptivity and hydraulic conductivity values reported as zero. With the exception of sample G, all hydrological calculations were also undertaken using only the infiltration data from after the recorded WDPT for each replicate (as opposed to the 50 s cut off) to determine if the inclusion of earlier data points altered the calculated values. No significant differences were found and the sorptivity and hydraulic conductivity values using the 50 s cut off value were used.

### Carbon and δ^13^C

After infiltration each core was air-dried to constant weight. The surface 5 mm was then manually removed using a finely adjustable sample stand that allowed the sample to be raised 5 mm above the alloy cylinder. The 0–5 mm section was ground to <0.5 mm and analysed for total C and δ^13^C using a Sercon IRMS at the University of New England, Armidale.

### Data Analysis

Percent crust cover, WDPT, sorptivity, hydraulic conductivity, porosity (averaged over both the 0–5 and 0.5–1 mm depth increments, see below for explanation of increment choice), C and δ^13^C (0–5 mm depth) were analysed for differences between crust types. General linear models were used with distributions and variable transformations specified as appropriate to meet the assumptions of each analysis. Where an analysis indicated a significant overall difference (at P<0.05), Tukey’s post-hoc tests were applied to determine pairwise differences between crust types.

The above analysis suggested potential associations between crust successional stage and the two variables of WDPT and hydraulic conductivity. Successional stage of crusts were transformed into a rank order variable (1 = Early, 2 = Mid, 3 = Mid to late, 4 = Late) and a general additive mixed model (GAMM) was used to model the association between the rank order of successional stage and both WDPT and hydraulic conductivity. Functional form of crust was specified as a random effect. The association was modeled using a spline with k = 3 degrees of freedom. Significance of the association was determined by plotting the modeled 95% confidence intervals at each successional stage. Summaries of model outputs can be found in Supporting Information (Table S1). Potential associations between pairs of variables were examined using scatterplots, correlations, and linear modelling. Porosity values were also combined into averages across different depth increments (100 µm increments to 5 mm depth) and analysed for significant pairwise associations with hydrological variables using scatterplots and correlation matrices. A strong association was found between hydraulic conductivity and porosity averaged across the 0–1 mm depth increment, and further data exploration indicated this association was due to the strong trend between hydraulic conductivity and the average porosity between 0.5 and 1 mm depth. For each significant association the strength of the association (R^2^) and significance (P) were determined from the appropriate linear model, and the results expressed as a linear regression. The potential confounding effect of different functional crust types on the significance of each linear association was additionally tested using a linear mixed model, specifying the crust type as a random effect. The proportional contribution of crust type to total model error was used as an indication of the strength of the effect of crust type in each analysis. Linear associations with 95% confidence intervals, accounting for the random effect off crust type, were produced using the approach of Fox [26]. Summaries of mixed linear model outputs can be found in Supporting Information (Table S2). Hydraulic conductivity and hydrophobicity values for sample G, the foliose lichen, were omitted from the regression analyses due to their extreme nature.

All data analysis was carried out in R version 2.14.1 [27] using the packages mgcv [28], lme4 [29], effects [26], and multcomp [30]. Significance was determined at the 0.05 level, and all assumptions of tests were met.

## Results

### Hydrological Values of Crust Types

**Figure 2:**
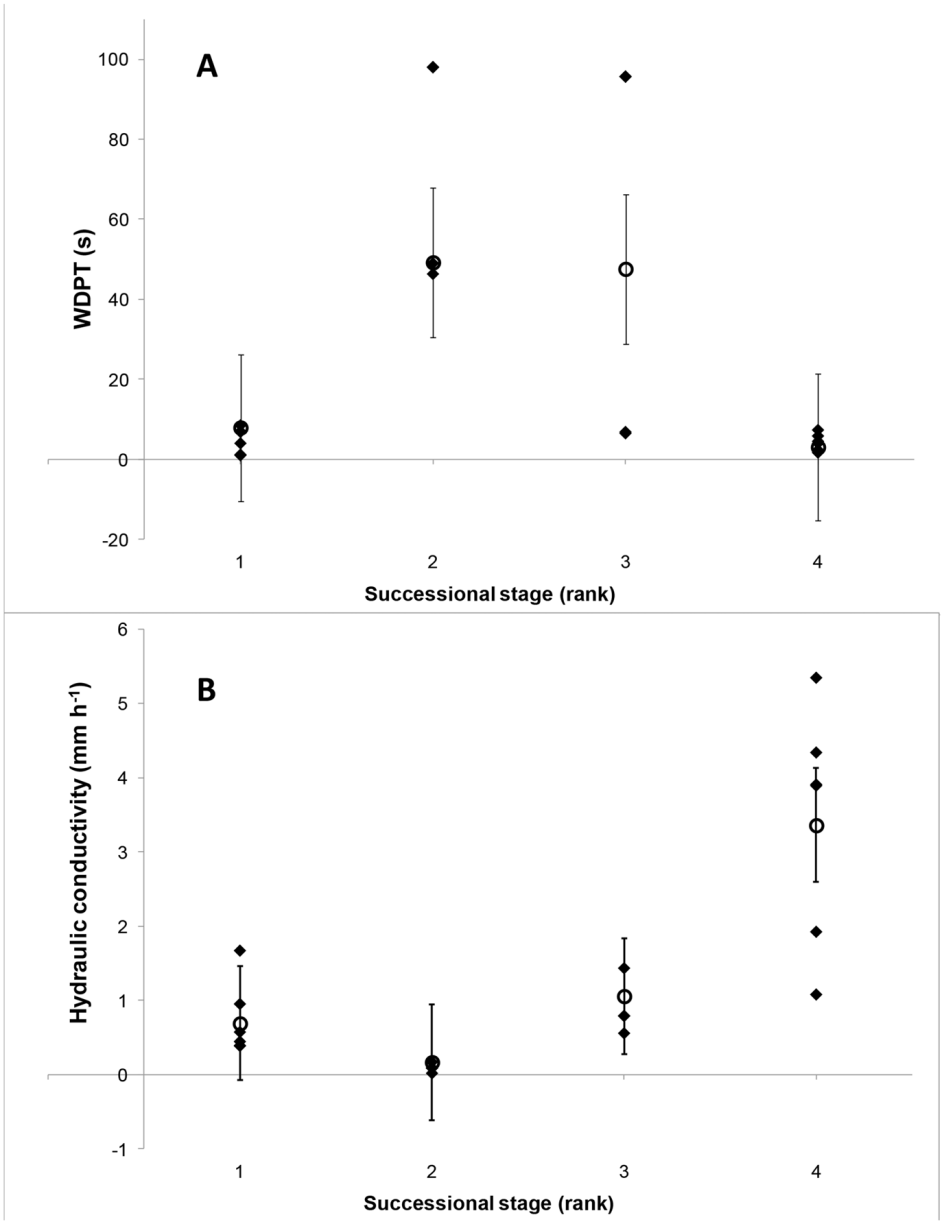
The general trend between successional stage of BSCs and hydrophobicity (as estimated from the results of the water drop penetration test) (A), and hydraulic conductivity (B). Successional stage is presented as a rank order variable (1 = Early, 2 = Mid, 3 = Mid to Late, 4 = Late). Open circles are predicted response and bars represent 95% confidence intervals following a GAMM analysis with 3 degrees of freedom. Black diamonds are individual data points.

Table 1 indicates the wide range of crust types sampled, both in functional form and using generally accepted classifications of BSC sensitivity to disturbance/ecological succession. There was high variation in WDPT values and hydraulic conductivity values within replicates of each crust type, yet there were still pronounced differences in the hydrophobic nature (as indicated by the WDPT) and the hydraulic conductivity of the crust types. The examined crusts spanned the range of hydrophilic to extremely hydrophobic behaviour according to common classifications of WDPT times [31]. The foliose lichen, considered as highly sensitive to disturbance and a late successional BSC, displayed an extreme degree of hydrophobicity and a corresponding zero value of hydraulic conductivity. The lowest degree of hydrophobicity (high hydrophilic behaviour) was shared between filamentous cyanobacteria, the liverwort, and the moss, spanning the sensitivity to disturbance/successional stage spectrum. The values of hydraulic conductivity ranged across 2 orders of magnitude, and with the exception of the foliose lichen, there was a significant (P = 0.006) trend of a ‘hump’ shaped association between hydrophobicity and crust classification (as sensitivity to disturbance or successional stage), and a similarly significant (P = 0.0001), but inverse humped association with hydraulic conductivity (Figure 2).

Sorptivity was low across all crust types (where it could be differentiated from zero), and no significant differences could be detected between the porosity of the crusts, either in the 0–1 or 0–5 mm depth intervals (Table 1). Carbon values in the 0–5 mm increment followed the sensitivity to disturbance classification in that crusts with low sensitivity to disturbance tended to have low C, compared with crusts with high sensitivity to disturbance. The δ ^13^C values did not show a consistent trend with crust successional stage/sensitivity to disturbance classification (Table 1).

### The Relationship between Hydrophobicity and Hydraulic Conductivity

**Figure 3:**
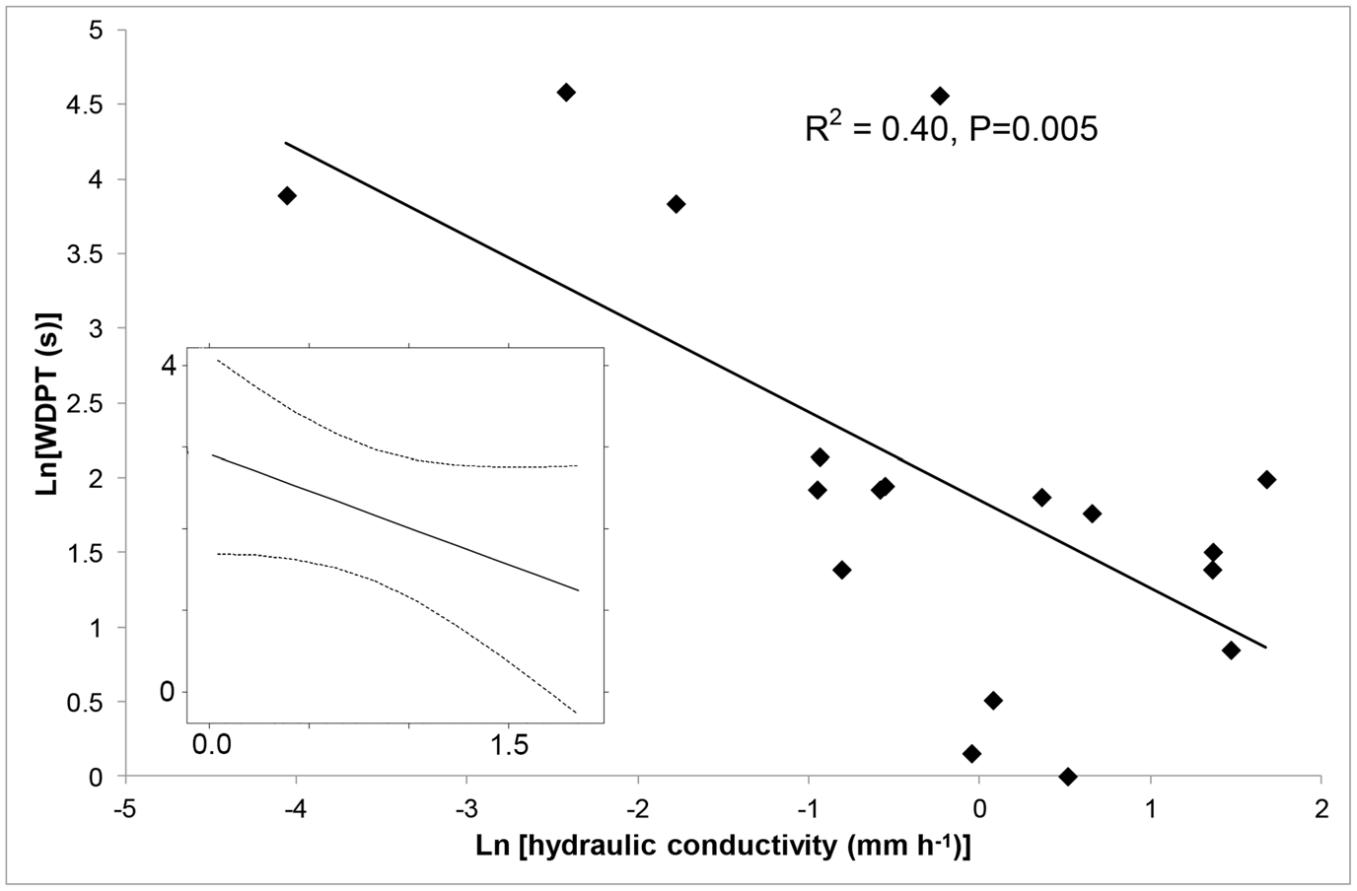
The relationship between hydraulic conductivity (mm h^−1^) and hydrophobicity (as estimated from the results of the water drop penetration test) of the crust surface. Values are expressed on the natural logarithm scale. INSET – the same association modeled with crust type as a random effect, showing the model prediction and 95% confidence intervals. This association was non-significant (P = 0.13). (see text for details). Values from sample G were omitted as outlined in text.

There was some indication of a relationship between hydrophobicity and hydraulic conductivity, but the association was confounded by crust type. Figure 3 shows the exponential decay relationship between hydraulic conductivity and the surface hydrophobicity (WDPT) across all samples, irrespective of crust type. The extremely low hydraulic conductivity values corresponded to WDPT times of greater than 20 seconds. Below this value the slope of the decay curve flattened out significantly, with a wider range of hydraulic conductivity values occurring for WDPT times less than approximately 8 seconds. However, when crust type was included as a random effect in the analysis this relationship was not significant (P = 0.13), and the random effect of crust type explained 52% of the total error in the model.

### The Relationship between Porosity and Biological Crust Cover

**Figure 4:**
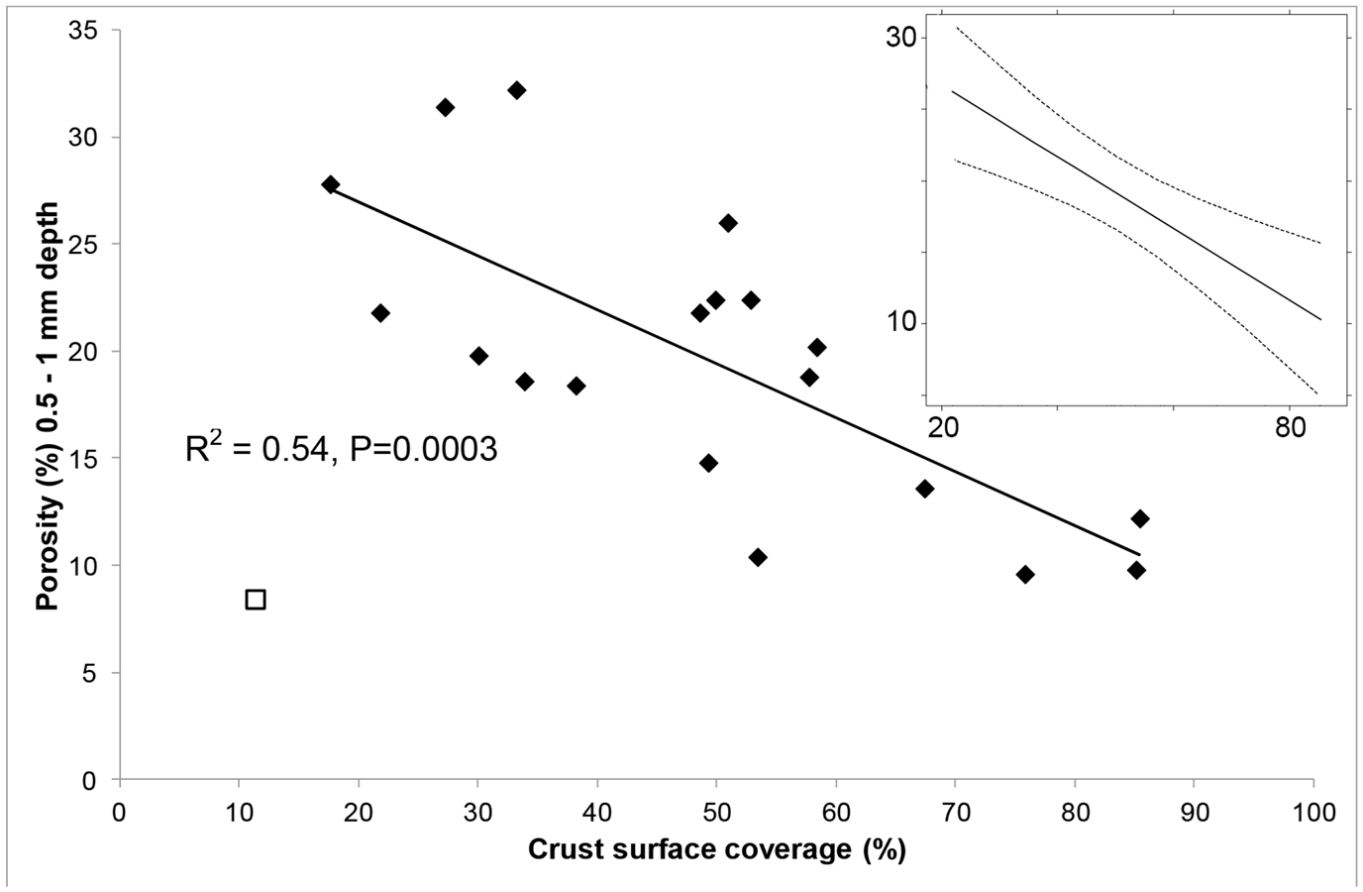
The relationship between surface coverage of BSC and the average porosity of the soil in the 0.5–1 mm depth interval. Open square symbol represents one replicate of sample B (filamentous cyanobacteria) which was omitted from the calculated relationship (see in text for details). The relationship is significant (R^2^ = 0.54, P = 0.0003). INSET – the same association modeled with crust type as a random effect, showing the model prediction and 95% confidence intervals. This association remained significant (P<0.0001) when crust type was included in the analysis as a random effect (see text for details).

Figure 4 depicts the only significant association detected between porosity and another variable. This association existed irrespective of the type of BSC (i.e. the association remained highly significant when crust type was specified as a random effect in the analysis (P = 0.0014). The random effect of crust type accounted for less than 1% of the total error in the model. Thus, irrespective of the type of BSC, as the percent of the sampled surface covered by the crust increased, the porosity averaged across the 0.5–1 mm depth increment decreased. Specifically, as the crust cover increased from approximately 20% of the surface to almost 90%, the porosity decreased by approximately 16%, from 27% to 11% (a relative decrease of 40%). Figure 4 also displays one outlier (open square symbol), which was omitted due to the difficulty in digitally thresholding and estimating the cover of the cyanobacteria in the sample.

### The Relationships between C, δ ^13^C and Hydraulic Conductivity

**Figure 5:**
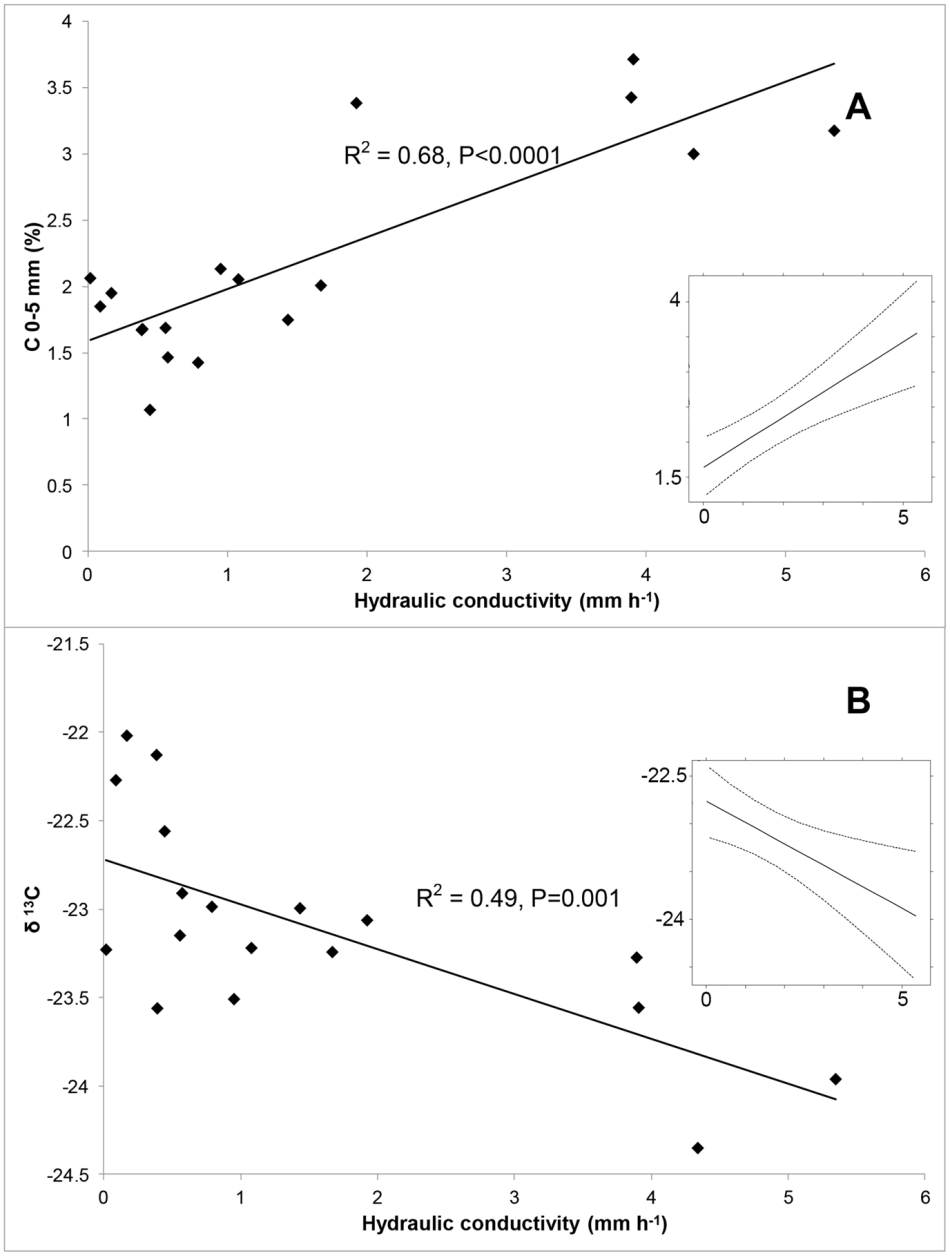
The relationships between C (%) in the 0–5 mm depth interval and hydraulic conductivity (mm h ^−**1**^ **) (A), and between δ ^13^C (0–5 mm) and hydraulic conductivity (mm h** ^−**1**^ **) (B).** Respective relationships are significant (R^2^ = 0.68, P<0.0001 and R^2^ = 0.49, P = 0.001). INSET – the same associations modeled with crust type as a random effect, showing the model prediction and 95% confidence intervals. These associations remained significant (P = 0.0005 and 0.0095 respectively) when crust type was included in the analyses as a random effect (see text for details). Values from sample G were omitted as outlined in text.

Figure 5a depicts the strong and highly significant relationship between C in the 0–5 mm depth increment and hydraulic conductivity, in which C in the crusted surface approximately doubled across the range of hydraulic conductivities measured. The random effect of crust type accounted for 43% of the total error in the model, but even accounting for this effect the association between C and hydraulic conductivity was still highly significant (P = 0.0005). As hydraulic conductivity increased and C likewise increased, the δ ^13^C signature became more negative (Figure 5b). This effect was also highly significant when the random effect of crust type was accounted for (P = 0.0095, with crust type accounting for 23% of the total error in the model).

## Discussion

There is a long history of conflicting reports of the links between soil surface hydrological behavior and the presence of BSCs. In particular the skew of data towards hot desert environments and the limited number of studies that cover a range of crust types have made it difficult to determine definitive relationships between BSCs and soil surface hydrology [2]. Many past studies have simply referred to BSCs as a single soil surface type, with little to no differentiation of crust types within a study [12]. At the same time, research has identified an ecological succession of BSCs that can occur in any given system. In a recent study Chamizo et al. [32] identified differences in infiltration between functionally different biological crusts types using a rainfall simulator. They found that late successional lichens had low infiltration but conversely moss had a very high infiltration. Our study supports these findings, as well as the existence of several trends suggested in previous meta-analyses [2], [7]. This is done by measuring several hydrological properties simultaneously at a very small scale, as opposed to the large scale measured via rainfall simulation.

All crust types were significantly variable in each measure. Thus, it was difficult to detect any general trends across the several crust types. What can be considered the extremes of early successional, low sensitivity to disturbance filamentous cyanobacteria, and the late successional, high sensitivity to disturbance liverwort and moss were easiest to delineate into two groups based on hydrological responses. Warren [7] states that hydrophobicity of BSCs is most commonly reported in relation to sandy soils, and with 70% sand, our findings support this. However, there was an extreme range of hydrophobicity between the crust types present at the site we have studied, from ‘hydrophilic’ to ‘severely hydrophobic’ in typical water drop penetration nomenclature [31]. The cyanobacteria were hydrophilic with low to medium hydraulic conductivity, and had low to medium C content. The late successional liverwort and moss were also hydrophilic but had relatively high hydraulic conductivities and a correspondingly high C content. The third crust type identified as being highly sensitive to disturbance was the extremely hydrophobic foliose lichen *Xanthoparmelia* spp. This crust was visually very fragile and exhibited a high C content but its hydrological behaviour was markedly different compared with all of the other crusts examined, in that it formed a practically water impermeable seal. These results suggest that hydrophobicity could be both species dependent (i.e. related to organism metabolites/polysaccharide secretions [7]) as well as being conditioned by factors such as functional classifications based on BSC morphology and disturbance response.

The functional crust types classified as having mid range sensitivity to disturbance exhibited a high degree of hydrophobicity and low hydraulic conductivity. Equating sensitivity to disturbance with ecological succession, there is a definite ‘hump’ shaped response in hydrological behaviour with succession. This response is most obvious in the hydrophilic-hydrophobic-hydrophilic nature of the BSCs, but is also clear (inverted) in the hydraulic conductivities. While these hump shaped associations were significant when the different crust types were included in the analysis as a random effect, our estimates of significance were based on limited degrees of freedom and our results only included a limited range of functional types at each successional stage. The higher hydraulic conductivities and lower hydrophobicities associated with the later successional stages also equate to the BSCs with pronounced above ground leaflet type structures (e.g. *Bryum* sp. (moss) and *Riccia* sp. (Liverwort)). It is possible that the structure of these above ground parts directly affects the hydrological behaviour of the surface at the scale we measured (i.e. water droplet scale). Similarly, the reduced above ground structures of the early to mid successional stage BSCs may account for their lower water affinity. The inclusion of other functional crust types from either the early, mid or late successional stages may alter the association we detected. Thus, while our data suggests a link between successional stage/functional form and hydrological behaviour, the link is not definitive and it is as yet unclear if there is a direct ecological benefit for BSCs in different successional stages or functional attributes to exhibit different hydrological behaviour.

Carbon of the BSCs appeared to lag behind the successional categories, only increasing significantly in the late successional BSCs. Porosity values do not appear to change significantly between crust types, but like other measurements, there was a wide range of variation between replicate measurements. Further exploration of this variation indicated several bivariate relationships that appeared to be independent of crust type.

The hump shaped pattern identified between BSC classification, hydrophobicity and hydraulic conductivities was present as a weak association between hydrophobicity and hydraulic conductivity, but this was confounded by crust type. Hydraulic conductivities of BSCs reported in the literature are often much higher than those reported here, but the difference is consistent with a downscaling effect when smaller samples such as aggregates are measured [33]. The largest average hydraulic conductivity in this study (*Bryum* spp.) was half of the lowest value found in arable Scottish soils using the same custom made infiltrometer. In that study, low values of hydraulic conductivity were considered an indicator of relatively high hydrophobicity. Our results do not directly support this previously found link, but it is probable that the low number of replicates may be compounding the random effect of crust type, effectively masking any existing relationship.

The BSCs with lower hydrophobicity and higher hydraulic conductivity also had the highest C. It is often postulated that high C associated with crusts may retard infiltration as organic detritus and exudates block pores [2]. While we did not find an association between crust coverage and C, we did find that higher crust coverage was associated with lower porosity just beneath the soil surface (i.e. the 0.5–1 mm increment of soil). The coarser scale at which we sampled C (the 0–5 mm increment due to practical limitations) compared to porosity may have masked any existing direct association between C and porosity. Alternatively, a relationship may exist in the pore size range below the resolution of our scans (<100 µm). Miralles-Mellado et al. [34] found several significant associations between porosity and organic C in BSCs from Spain, in which they measured porosity to 50 µm resolution. Thus, both of these possibilities need further examination, particularly as the links between different crust types and micro- and macroporosity do not appear to have been examined in previous BSC studies [14]. Either a smaller volume of the crusted surface could be CT scanned or a larger detector system could be employed to increase the resolution of 3-dimensional scanning and hence the range of pore sizes detected [35]. The latter of these two options is preferable, as selecting a smaller sample volume will decrease the likelihood of calculating a representative porosity for a given increment of soil. Despite these limitations, our results support the hypothesis that higher crust coverage ‘clogs’ larger pores and reduces associated porosity.

The strong relationship between surface C and hydraulic conductivity may indicate one of two mechanisms; either the increase in hydraulic conductivity associated with the late successional crusts we examined changes wetting and drying cycles and increases C over time, both through crust biomass and exudates/decomposition, or these crusts tend to develop after a surface has increased C to a point that the hydrological properties are altered (i.e. a mid successional crust that has existed for long enough to slowly accrue C to a point that hydraulic conductivity is affected, initiating a change in crust type). Which of these cause and effect relationships dominate needs to be determined to develop our understanding of how, and when, BSCs can be associated with higher surface C. Examining this relationship may also provide information on the ecological benefits both early and late successional BSCs may derive from their relatively hydrophilic nature compared with mid successional crusts.

The link between C and hydrological properties of the BSCs is further evidenced by the association between the isotopic signature of C in the different surfaces and hydraulic conductivity. Biological crusts with the highest hydraulic conductivities also exhibited the most negative δ ^13^C values. As non-vascular plants are expected to show less C isotopic fractionation with a higher frequency of saturation, this indicates that the BSCs with the lowest inherent ability to conduct water were also those that were saturated with water the longest in this semi-arid environment. While this feature of non-vascular plants has been examined in other environments [11], [15], to our knowledge this is the first time this trend has been examined in the type of rangeland system we have investigated. These results suggest that the physiological response (C acquisition) of the different crusts is affected by the hydrological condition of the surface, which in turn alters the chemical signature of the surface. There may be potential to extend the use of C isotopes in this type of research to the occurrence of previous inundation of semi-arid and arid sites, or spatial dynamics of inundation without the need to observe flooding first hand. In addition, given the widespread nature of BSCs in these types of environments, this secondary form of isotopic fractionation may necessitate a re-examination of some model outputs that use δ ^13^C soil signatures to model shifts between C3 and C4 vegetation in these types of environments [36], [37].

### Conclusions

This is the first time various hydrological characteristics of a range of crust types have been examined at one site at such a small scale. The high variability found within each crust type and between each crust type helps explain the myriad of different hydrological responses reported in the literature to date. These findings also emphasise the danger of relying on a broad classification such as ‘BSC’ in which to measure hydrological responses, when a range of crust types, proportional surface coverages, and variable responses may exist at any one site. We did not find any linear relationship between disturbance/successional classification of BSCs and hydrological properties, but there was evidence of a ‘hump’ shaped association, in that the mid successional/mid range disturbance sensitive crust types were more impermeable to water than either the early pioneering or late successional functional forms, with the exception of *Xanthoparmelia* spp., a highly fragile late successional crust that exhibited higher water impermeability than any other crust type. This trend needs to be confirmed in a wider range of crust types within the spectrum of functional classification.

The small scale at which our measurements were undertaken has allowed us to determine some of the ‘inherent’ hydrological characteristics of functionally different BSCs, which may serve studies that go on to examine hydrological responses of crusted surfaces at larger scales. For example, the BSCs with the higher hydraulic conductivities and higher values of C in this study are those associated with more pronounced surface relief and tortuosity at larger scales [5], all of which would combine to influence the hydrological responses seen at these larger scales. Similarly, the high runoff associated with smooth, cyanobacteria dominated areas may not be associated with hydrophobicity, but may be related to other factors such as high surface coverage and corresponding low porosity. However, such upscaling will not be simple – BSCs occur as complex assemblages of functional types, and an amalgamated response may be difficult to relate back to small scale measurements, unless only one or two functional forms are dominant at the large as well as small scale. One additional factor related to upscaling responses needs further examination – the influence of hydrological behaviour of crusted surfaces on C isotopic signatures of soil surfaces. As semi-arid and arid rangelands can have large areas dominated by BSCs, the effect of crust isotopic signatures on vegetation change modelling needs to be assessed.

## Supporting Information

Figure S1 **Image of the study site showing mixed mid-storey of** ***Geijera*** **,** ***Dodonaea*** **and** ***Eremophila*** **sp and minimal herbaceous ground cover.**
(TIF)

Table S1 Summarised general additive mixed model (GAMM) outputs for the relationships depicted in Figure 2, including crust type as a random factor, and specifying k = 3 degrees of freedom for the non-parametric spline term of the model.
(DOCX)

Table S2 Summarized linear mixed model outputs for bivariate regressions including crust type as a random factor, including MCMC (Markov Chain Monte Carlo) estimated P values.
(DOCX)
