# Supplementary figures and images for: Ecological Succession, Hydrology and Carbon Acquisition of Biological Soil Crusts Measured at the Micro-Scale
Source: PLoS One. 2012 Oct 30;7(10):e48565. doi: 10.1371/journal.pone.0048565 (PMC3484118; doi:10.1371/journal.pone.0048565)

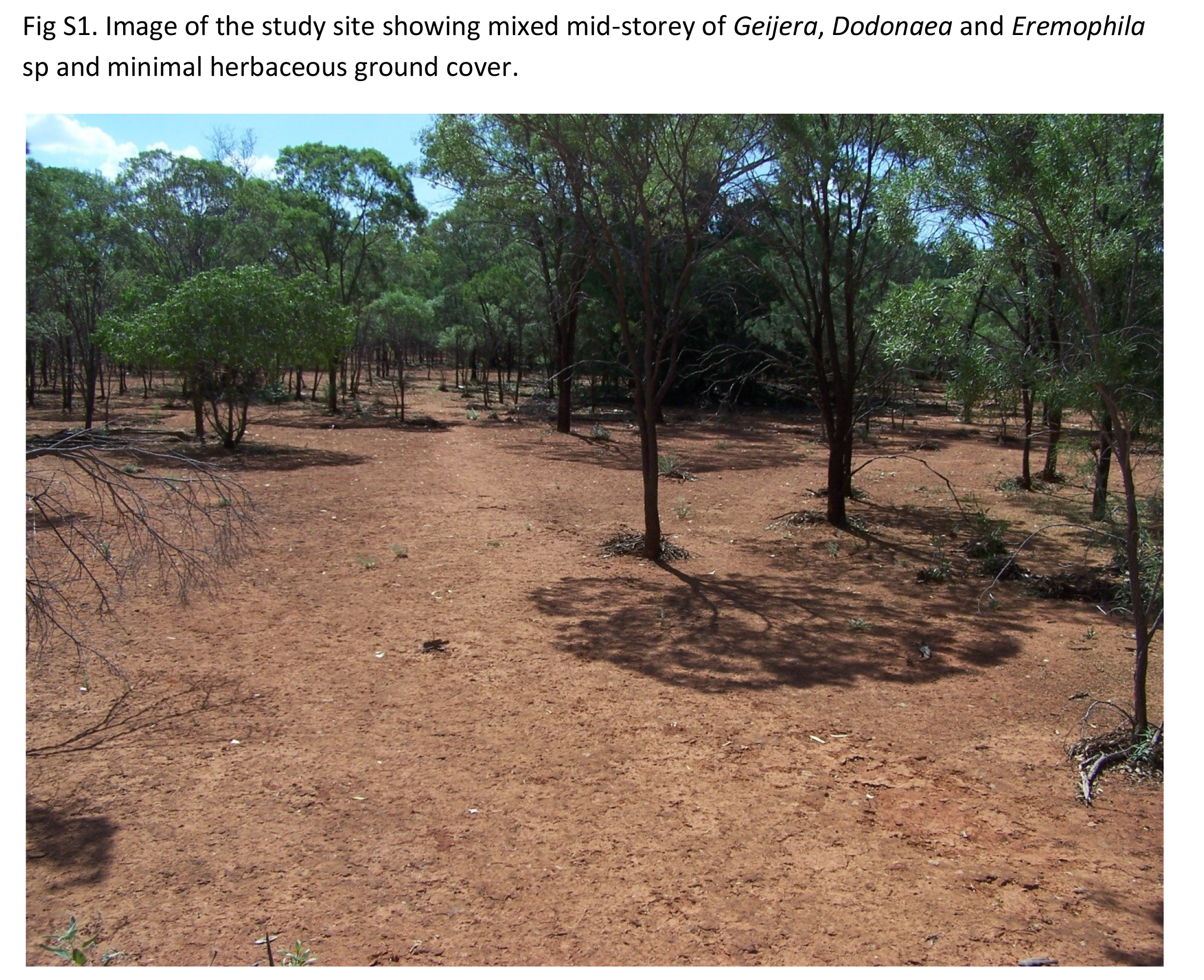

Supplement: Figure S1 — Image of the study site showing mixed mid-storey of Geijera , Dodonaea and Eremophila sp and minimal herbaceous ground cover. (TIF) [file pone.0048565.s001.tif]
