# Supplementary material for: Ecological Succession, Hydrology and Carbon Acquisition of Biological Soil Crusts Measured at the Micro-Scale
Source: PLoS One. 2012 Oct 30;7(10):e48565. doi: 10.1371/journal.pone.0048565 (PMC3484118; doi:10.1371/journal.pone.0048565)
Supplement: Table S1 — Summarised general additive mixed model (GAMM) outputs for the relationships depicted in Figure 2, including crust type as a random factor, and specifying k = 3 degrees of freedom for the non-parametric spline term of the model. (DOCX) [file pone.0048565.s002.docx]

Table S1. Summarised general additive mixed model (GAMM) outputs for the relationships depicted in Figure 2, including crust type as a random factor, and specifying k=3 degrees of freedom for the non-parametric spline term of the model.

| Model 1 – WDPT (s) ~ spline(succession order, degrees of freedom=3) | | | | |
| --- | --- | --- | --- | --- |
| Parametric coefficients | | | | |
| Term | Estimate | Std error | t-value | P-value |
| Intercept | 19.81 | 5.31 | 3.73 | 0.002 |
| Approximate significance of smooth terms | | | | |
| Term | edf | Ref.df | F | P-value |
| spline(succession order) | 1.94 | 1.94 | 7.40 | 0.006 |
| R^2^ (adjusted) 0.45 | |  |  |  |
| Model 2 - Hydraulic conductivity (mm h^-1^)~ spline(succession order, degrees of freedom=3) | | | | |
| Parametric coefficients | | | | |
| Term | Estimate | Std error | t-value | P-value |
| Intercept | 1.55 | 0.31 | 5.09 | 0.0001 |
| Approximate significance of smooth terms | | | | |
| Term | edf | Ref.df | F | P-value |
| spline(succession order) | 1.84 | 1.84 | 7.26 | 0.007 |
| R^2^ (adjusted) 0.65 | |  |  |  |
