# Supplementary material for: Ecological Succession, Hydrology and Carbon Acquisition of Biological Soil Crusts Measured at the Micro-Scale
Source: PLoS One. 2012 Oct 30;7(10):e48565. doi: 10.1371/journal.pone.0048565 (PMC3484118; doi:10.1371/journal.pone.0048565)
Supplement: Table S2 — Summarized linear mixed model outputs for bivariate regressions including crust type as a random factor, including MCMC (Markov Chain Monte Carlo) estimated P values. (DOCX) [file pone.0048565.s003.docx]

Table S2. Summarized linear mixed model outputs for bivariate regressions including crust type as a random factor, including MCMC (Markov Chain Monte Carlo) estimated P values.

| **Model 1 – log WDPT (s) ~ log (Hydraulic conductivity (mm h^-1^))** | | | | |
| --- | --- | --- | --- | --- |
| Random effects | | |  |  |
| Groups | Variance | std dev |  |  |
| Crust type | 0.66 | 0.81 |  |  |
| Residual | 0.62 | 0.78 |  |  |
| Observations (groups) | 18 (6) |  |  |  |
| Fixed effects | | | | |
|  | Estimate | std error | t-value | P-value |
| Intercept | 2.91 | 0.58 | 5.06 | 0.0001 |
| log (Hydraulic conductivity) | -0.90 | 0.57 | -1.59 | 0.13 |
| Correlation of Fixed Effects: -0.75 | | |  |  |
| **Model 2 – Porosity (%) 0.5-1mm depth ~ cover (%)** | | | | |
| Random effects | | |  |  |
| Groups | Variance | std dev |  |  |
| Crust type | 1.31e-09 | 3.61e-05 |  |  |
| Residual | 2.47e+01 | 4.97e+00 |  |  |
| Observations (groups) | 17 (6) |  |  |  |
| Fixed effects | | | | |
|  | Estimate | std error | t-value | P-value |
| Intercept | 31.7 | 3.49 | 9.09 | <0.0001 |
| cover | -0.25 | 0.06 | -3.91 | 0.0014 |
| Correlation of Fixed Effects: -0.94 | |  |  |  |
| **Model 3 – Carbon (%) (0-5mm depth~ Hydraulic conductivity (mm h^-1^)** | | | | |
| Random effects | | |  |  |
| Groups | Variance | std dev |  |  |
| Crust type | 0.10 | 0.31 |  |  |
| Residual | 0.13 | 0.36 |  |  |
| Observations (groups) | 18 (6) |  |  |  |
| Fixed effects | | | | |
|  | Estimate | std error | t-value | P-value |
| Intercept | 1.64 | 0.20 | 8.16 | <0.0001 |
| Hydraulic conductivity | 0.36 | 0.08 | 4.30 | 0.0005 |
| Correlation of Fixed Effects: -0.64 | |  |  |  |
| **Model 4 – δ^13^Carbon ~ Hydraulic conductivity (mm h^-1^)** | | | | |
| Random effects | | |  |  |
| Groups | Variance | std dev |  |  |
| Crust type | 0.05 | 0.22 |  |  |
| Residual | 0.16 | 0.40 |  |  |
| Observations (groups) | 18 (6) |  |  |  |
| Fixed effects | | | | |
|  | Estimate | std error | t-value | P-value |
| Intercept | -22.76 | 0.17 | -128.93 | <0.0001 |
| Hydraulic conductivity | -0.22 | 0.08 | -2.94 | 0.0095 |
| Correlation of Fixed Effects: -0.67 | |  |  |  |
